# Non-cognitive characteristics predicting academic success among medical students in Sri Lanka

**DOI:** 10.1186/1472-6920-12-66

**Published:** 2012-08-03

**Authors:** Priyanga Ranasinghe, Amaya Ellawela, Saman B Gunatilake

**Affiliations:** 1 University Medical Unit, Colombo South Teaching Hospital, Kalubowila, Sri Lanka; 2 Faculty of Medical Sciences, University of Sri Jayewardenepura, Sri Jayewardenepura, Sri Lanka

**Keywords:** Academic performance, Predictors, Non-cognitive characteristics, Medical students, Sri Lanka

## Abstract

**Background:**

To identify non-cognitive and socio-demographic characteristics determining academic success of Sri Lankan medical undergraduates.

**Methods:**

A retrospective study among 90 recently graduated students of the Faculty of Medical Sciences, University of Sri Jayewardenepura, Sri Lanka. Students were stratified into two equal groups; ‘High-achievers’ (honours degree at the final MBBS examination) and ‘Low-achievers’ (repeated one or more subjects at the same examination). A revised version of the Non-cognitive Questionnaire (NQ) with additional socio-demographic data was the study instrument. Academic performance indicator was performance at the final MBBS examinations. A binary logistic regression analysis was performed using the dichotomous variable ‘Honours degree at final MBBS’ as the dependant factor.

**Results:**

Males were 56.7%. Mean age ± SD was 26.4 ± 0.9 years. ‘High-achievers’ were significantly younger than ‘Low-achievers’. Significant proportion of ‘High-achievers’ were from the Western province and selected to university from Colombo district. A significant majority of ‘High-achievers’ entered medical school from their first attempt at GCE A/L examination and obtained ‘Distinctions’ at the GCE A/L English subject. ‘High-achievers’ demonstrated a significantly higher mean score for the following domains of NQ; Positive self-concept and confidence, realistic self-appraisal, leadership, preference of long range goals and academic familiarity.

The binary logistic regression indicates that age, being selected to university from Colombo district, residency in Western province, entering university from GCE A/L first attempt, obtaining a ‘Distinction’ for GCE A/L English subject, higher number of patient-oriented case discussions, positive self-concept and confidence, leadership qualities, preference of long range goals and academic familiarity all significantly increased the odds of obtaining a Honours degree.

**Conclusion:**

A combined system incorporating both past academic performance and non-cognitive characteristics might help improve the selection process and early recognition of strugglers.

## Background

Medicine is a complex and demanding field of study, medical undergraduates not only requires skill and competence in multiple disciplines, they also need to posses the ability to acquire knowledge on a wide-range of subjects over short-period of time. Medical schools worldwide use different methodologies and criteria to select ‘ideal’ candidates, which include cognitive factors like previous academic performance and non-cognitive factors like personality, performance at interview, comments by referees, personal statements in forms of essays and assessing the involvement in extracurricular activities. However selecting the “ideal” students has always been a challenge and a subject of much debate [1-3]. While traditional medical schools usually tend to depend solely on higher previous academic performance, most modern medical schools are beginning to incorporate a set of non-cognitive selection criteria [4,5]. A medical student’s training represents a major investment by the government and a considerable personal investment of time and money for the student. Thus, in addition to selecting the ‘ideal’ students it is also important to minimise the rate of attrition by having robust mechanisms to identify and support those who are “struggling”.

Each year nearly seven hundred students graduate from the seven medical faculties in Sri Lanka. The student selection at present is solely based on performance at the GCE Advanced Level examination. The government schools where students study for this examination are of two types 1) National schools – supervised by the central government and has better facilities; 2) Provincial schools – supervised by the respective provincial councils and usually has lesser facilities. Due to this disparity in educational facilities a district based merit system is used for selection [6]. The vacancies for medicine at the universities are filled according to the following 3 sets of criterion: a) All Island Merit criteria: Up to 40% of the available vacancies will be filled in order of Z-Scores for the GCE A/L examination ranked on an all island basis, b) District Merit criteria: Up to 55% of the available places will be allocated to the 25 administrative districts in proportion to the total population, that is, on the ratio of the population of the district concerned to the total population of the country and c) A special allocation up to 5% of the vacancies will be allocated to 16 educationally disadvantaged districts (does not include Colombo) [6]. Furthermore, to be eligible for selection under this criterion, a student must obtain at least a ‘Simple’ pass for each of the three GCE A/L subjects. Recent studies have demonstrated that this sole admission criterion is a poor predictor of academic success of Sri Lankan medical students [7,8].

The students at Faculty of Medical Sciences, University of Sri Jayewardenepura undergo a five year training programme. The basic sciences of Anatomy, Biochemistry and Physiology are taught in the first two years followed by a summative examination in the second year (commonly known as 2nd MBBS examination), which students must pass in order to proceed to the next level of training. Third and fourth year curricula include training in the para-clinical or applied science subjects, together with clinical training in hospitals. Finally, in the final year students are trained on the clinical subjects of Medicine, Surgery, Obstetrics and Gynaecology, Paediatrics and Psychiatry which is followed by the final examination (commonly known as the final MBBS examination) for graduation which includes multiple choice question paper (MCQ), structured essay question paper (SEQ) and a clinical examination in each subject.

There are no studies examining the non-cognitive predictors of academic performance of medical undergraduates. The primary objective of the present study is to identify socio-demographic and other non-cognitive characteristics determining academic success of medical undergraduates at Faculty of Medical Sciences, University of Sri Jayewardenepura, Sri Lanka.

## Methods

### Study setting

This retrospective study was conducted among medical students of the Faculty of Medical Sciences, University of Sri Jayewardenepura, Sri Lanka in July 2010 to June 2011. Ninety undergraduates who completed the final MBBS examination in 2010 and 2011 were invited for the study. The students were stratified in to two equal groups; Group ‘A’ (‘High achievers’) included students who have obtained a honours degree at the final MBBS examination, while Group ‘B’ (‘Low achievers’) included students who have referred in one or more subjects at the final MBBS examination. Ethical clearance for the study was obtained from the Ethics Review Committee, Faculty of Medical Sciences, University of Sri Jayewardenepura, Sri Lanka.

### Study instrument

A revised version of the Non-cognitive Questionnaire (NQ) [5] was used as the study instrument. The NQ was developed by Sedlacek and colleagues at University of Maryland as a self-assessment tool that explores non-cognitive domains associated with undergraduate academic success. Items pertaining directly to college students or to the University of Maryland were changed to refer to medical students (eg, "My friends and relatives don't feel I should go to college" was changed to "My friends and relatives don't feel I should go to medical school," or "I am as skilled academically as the average applicant to University of Maryland" was changed to "I am as skilled academically as the average applicant to medical school"). In addition socio-demographic data including age, gender, attempt at GCE A/L, district of selection to medical school, province of residence, educational background of parents, competency in English, availability of books and other facilities and attendance to lectures were also collected. The academic performance indicator was the student performance at the 2^nd^ MBBS and final MBBS examinations. This self-administered questionnaire was distributed amongst the invited students by e-mail or by asking them to collect the questionnaire in person from the Department of Clinical Medicine at the university.

### Statistical analysis

All data were double-entered and cross checked for consistency. Data were analysed using SPSS version 14 (SPSS Inc., Chicago, IL, USA) statistical software package. The significance of the differences between proportions (%) and means were tested using z-test and Student’s t-test or ANOVA respectively.

The Faculty of Medical Sciences at the University of Sri Jayewardenepura is situated in the ‘Colombo’ district in the ‘Western’ province of Sri Lanka. Hence for analytical purposes the districts and provinces were divided in to two groups ‘Colombo district’ or ‘Other districts’ and ‘Western province’ or ‘Other provinces’. A binary logistic regression analysis was performed using the dichotomous variable ‘Honours degree at final MBBS’ as the dependant factor (0 = No; 1 = Yes) and age, gender (0 = Male, 1 = Female), GCE A/L attempt to enter medical school (0 = Second/Third attempt, 1 = First attempt), district of selection to university (0 = Not Colombo, 1 = Colombo), Province of residence (0 = Non-Western, 1 = Western), grade for GCE A/L English subject (0 = Credit, 1 = Distinction), number of patient-oriented case discussions and score for the seven domains of the NQ (Positive self-concept and confidence, Realistic self-appraisal, Availability of support person, Leadership, Long range goals, Ability to establish community ties, Academic familiarity) as independent variables. In all statistical analyses a p value < 0.05 was considered significant.

## Results

Sample size was 90, with 56.7% being males. Mean age (±SD) was 26.4 ± 0.9 years. Socio-demographic characteristics of the two groups ‘High achievers’ and ‘Low achievers’ are summarized in Table 1. ‘High achievers’ were significantly younger than ‘Low achievers’ (p < 0.05). There was no significant gender or ethnicity difference between the two groups. A statistically significant proportion of ‘High achievers’ were from the Western province and was selected to the university from the Colombo district (Table 1).

**Table 1:** Socio-demographic characteristics of ‘High achievers’ and ‘Low achievers’

	**High achievers**	**Low achievers**
Mean age (±SD)*	26.0 (±0.9)	26.7 (±0.7)
Gender		
Male	27 (60.0%)	24 (53.3%)
Female	18 (40.0%)	21 (46.7%)
Ethnicity		
Sinhalese	39 (86.6%)	41 (91.1%)
Tamil	3 (6.7%)	3 (6.7%)
Moor	3 (6.7%)	1 (2.2%)
District of Selection*		
Colombo	31 (68.9%)	11 (24.4%)
Other	14 (31.1%)	34 (75.6%)
Province of residence*		
Western	37 (82.2%)	20 (44.4%)
Other	8 (17.8%)	25 (55.6%)

*p <0.05.

Eighty four percent of the ‘High achievers’ entered university by sitting for GCE A/L examinations at a National School, while this number was only 37.8% for the ‘Low achievers’ (p < 0.05) and a significant majority (60.0%) of ‘Low achievers’ were from Provincial schools. A statistically significant majority (62.2%) of ‘High achievers’ entered medical school from their first attempt at GCE A/L examination, however 88.9% of the ‘Low achievers’ entered medical school either in the 2^nd^ or 3^rd^ attempts. There was no statistically significant difference between the two groups when considering the highest educational qualifications of the either parents (Table 2). Eighty two percent of ‘High achievers’ felt that their knowledge in English language was either ‘excellent’ or ‘good’, and a majority (71.1%) of ‘Low achievers’ felt that their knowledge is either only ‘fair’ or ‘poor’. This was also reflected in their performance at the GCE A/L English subject where a statically significant majority of ‘High achievers’ obtained ‘Distinctions’ (Table 2). A statistically significant majority (60.0%) of ‘Low achievers’ felt that their performance was adversely affected by poor English language skills, while only 13.3% ‘High achievers’ felt the same. In addition over 95% of ‘High achievers’ were involved in extra-curricular activities during school period when compared to only 42.4% in the ‘Low achievers’ group.

**Table 2:** Educational background (school level) of the two groups

	**High achievers (%)**	**Low achievers (%)**
School attended*		
National school	38 (84.5%)	17 (37.8%)
Provincial school	5 (11.1%)	27 (60.0%)
Private school	2 (4.4%)	1 (2.2%)
GCE (A/L) attempt to enter medical school*		
1^st^ attempt	28 (62.2%)	5 (11.1%)
2^nd^ attempt	13 (28.9%)	24 (53.3%)
3^rd^ attempt	4 (8.9%)	16 (35.6%)
Highest academic qualification of either parent		
Primary education	1 (2.2%)	1 (2.2%)
Secondary education	15 (33.3%)	25 (55.6%)
Tertiary education	29 (64.5%)	19 (42.2%)
GCE (A/L) English subject performance*		
Distinction	31 (68.9%)	6 (13.3%)
Credit pass or below	14 (31.1%)	39 (86.7%)
Involved in extra-curricular activities*	44 (97.8%)	19 (42.2%)

*p < 0.05.

Students’ performance during the undergraduate period was assessed by evaluating the result obtained at the 2^nd^ MBBS examination. A significant majority (84.4%) of ‘High achievers’ have obtained an ‘Honours’ pass for this examination (1^st^ Class/2^nd^ Upper or Lower division), while ‘Low achievers’ have either obtained a simple pass (33.0%) or repeated (64.4%) the exam. When it came to extra-curricular activities during the undergraduate period there was no significant difference between the two groups (Table 3). In the ‘High achievers’ group students attended lectures during undergraduate period selectively (64.4%), and most ‘Low achievers’ attended all lectures regularly (60.0%). The mean number (±SD) of patient-oriented case discussions with seniors (Consultants/Senior registrars/Registrars) prior to the final MBBS exam was significantly (p < 0.001) higher for ‘Achievers’ (18.5 ± 12.7) than ‘Low achievers’ (11.4 ± 8.1). Sixty eight percent of ‘Low achievers’ felt that their parents and relative being far away (i.e. studying far away from home) affected their performance at medical school, however the same was said by only 15.6% of ‘High achievers’. Among the ‘High achievers’ 68.9% had a personnel computer, in comparison to only 28.9% of ‘Low achievers’ (p < 0.001). In addition 53.3% of ‘High achievers’ had full time access to internet, while only 22.2% of ‘Low achievers’ had the same (p < 0.001).

**Table 3:** **Students’ performance at 2**^**nd**^**MBBS, lecture attendance and computer access**

	**High achievers (%)**	**Low achievers (%)**
Performance at 2^nd^ MBBS examination*		
Honours pass	38 (84.4%)	1 (2.2%)
Simple pass	4 (8.9%)	15 (33.3%)
Repeated	3 (6.7%)	29 (64.0%)
Attendance to lectures*		
Selectively	29 (64.4%)	18 (40.0%)
Non-selectively (all)	16 (35.9%)	27 (60.0%)
Computer access*		
Personnel computer	31 (68.9%)	13 (28.9%)
Limited access only	14 (31.1%)	32 (71.1%)
Internet access*		
Full time	24 (53.3%)	10 (22.2%)
Limited access	21 (46.7%)	35 (87.8%)
Involved in extra-curricular activities*	29 (64.4%)	19 (42.2%)

*p < 0.05.

The mean scores obtained by the two groups for the seven domains of the Non-cognitive questionnaire are displayed in Table 4. The ‘High achievers’ demonstrated a significantly higher mean score for the domains of; Positive self-concept and confidence, realistic self-appraisal, leadership, preference of long range goals and academic familiarity (p < 0.05).

**Table 4:** Score for the Non-cognitive questionnaire of the two group

	**Mean score (±SD)**	
	**High achievers**	**Low achievers**
Positive self-concept and confidence*	18.6 (±3.5)	13.8 (±2.9)
Realistic self-appraisal*	10.4 (±1.8)	8.6 (±1.5)
Availability of support person	11.4 (±2.2)	10.2 (±1.6)
Leadership*	7.5 (±2.7)	5.7 (±1.6)
Long range goals*	12.1 (±3.5)	7.5 (±2.2)
Ability to establish community ties	4.1 (±1.8)	3.1 (±1.3)
Academic familiarity*	4.3 (±2.8)	1.8 (±1.7)

*p < 0.05.

The results of the binary logistic regression analysis using the dichotomous variable ‘Honours degree at final MBBS’ (0 = No, 1 = Yes) as the dependant factor and other independent variables mentioned above are shown in Table 5. The overall model was statistically significant as determined by the likelihood ratio test (χ^2^ = 38.22, p < 0.05). The Cox & Snell R-Square and Nagelkerke R Square values were 0.634 and 0.845 respectively. Hosmer and Lemeshow goodness-of-fit statistic (χ^2^) was 24.34 and suggested that the model was fit for the given data. The results indicate that age, being selected to university from Colombo district, residency at Western province, entering university from GCE A/L first attempt, obtaining a ‘Distinction’ for GCE A/L English subject, higher number of patient-oriented case discussions, positive self-concept and confidence, leadership qualities, preference of long range goals and academic familiarity all significantly increased the odds of obtaining a Honours degree at the Final MBBS.

**Table 5:** Logistic regression analyses of ‘Honours degree at final MBBS’ & measured parameters

	**β (SE)**	**Wald statistic**	***p* value**
Age	−0.532 (2.476)	0.046	<0.05
Gender			
Male (reference category)			
Female	−1.668 (1.511)	1.219	NS*
District of selection to university			
Not-Colombo (reference category)			
Colombo	1.816 (2.316)	1.124	<0.05
Province of residence			
Non-Western (reference category)			
Western	1.536 (2.521)	1.129	<0.05
GCE A/L attempt			
Second or Third (reference category)			
First	12.743 (5.194)	6.020	<0.05
GCE A/L English subject			
Credit (reference category)			
Distinction	2.794 (1.526)	3.354	<0.05
Patient-oriented case discussions	0.691 (0.290)	5.692	<0.05
Non-Cognitive Questionnaire domains			
Positive self-concept and confidence	1.858 (1.372)	5.324	<0.05
Realistic self-appraisal	0.477 (0.673)	0.502	NS*
Availability of support person	0.718 (0.485)	2.193	NS*
Leadership	1.533 (0.927)	2.731	<0.05
Long range goals	1.805 (1.455)	3.124	<0.05
Ability to establish community ties	0.065 (0.681)	0.014	NS*
Academic familiarity	3.125 (1.277)	5.987	<0.05

*NS – Not significant.

## Discussion

Medicine is considered to be the longest and most stressful course of undergraduate study [9]. This is the first study from Sri Lanka that evaluates the effects of non-cognitive characteristics on medical student’s success or failure. Our results suggest that past academic performance is not the sole-predictor of future academic success among Sri Lankan medical student. In Sri Lanka students are selected to all seven medical schools depending on their performance at the GCE A/L examination. We demonstrate that students gaining university entrance from 2^nd^ or 3^rd^ attempts performed poorly in comparison to 1^st^ attempt students. In addition entering university in the 1^st^ attempt also increased the odds of obtaining a ‘Honours’ degree at the final MBBS examination. Similar findings have been demonstrated by two different authors from Sri Lanka [7,8]. Therefore serious consideration must be given about revising the admission criteria to medical schools and restricting the number of attempts by a student at GCE A/L examination to gain university admission.

Due to the disparity in educational facilities in Sri Lankan government schools a district based merit system is used for students selection, where the marks obtained by a student for the three subjects at the GCE A/L is converted to a single standardized Z-Score. Each district is then given a cut-off Z-Score for selection, where districts with lower facilities having a lower Z-Score and vice versa [7]. Our results demonstrate that being from the Western province and being selected to university from Colombo district were both significant predictors of academic success. Colombo being the commercial district of the country has the highest population density and the best facilities. Thus, the GCE A/L cut-off for the Colombo district is invariably significantly higher than for the other districts resulting in a higher competition among Colombo students. This competition invariably results in only the very best students getting selected from Colombo and this might be a probable reason for their ultimate academic success. A downside of this selection process is that it results in many talented students from the Colombo district and sub-urban areas not gaining entry into medical school. Whereas students with a much lower mark at the same entry exam from underprivileged districts enter medical school. Since rapid urbanization and improvement living standards have occurred in many areas of Sri Lanka over the past decades, there is a need re-define underprivileged areas of the country.

The students’ competency in English as assessed by performance at the GCE A/L examination English subject was also a significant predictor of success. In all seven Sri Lankan medical schools teaching and examinations including case discussions are conducted solely in English. However, Sinhalese is the mother-tongue of nearly 90% of Sri Lankans [10] and until recently the GCE A/L examination was conducted only in Sinhalese. Students less competent in English face obstacles when learning and performing at examinations. The poor knowledge in English also hinders participation in academic activities such as case discussions. Our results also demonstrate that under achieving student take part less in patient oriented case discussion when compared to the successful ones. Students who are from Colombo district being the commercial capital and having the highest number of English speaking population has the language advantage also in addition to better facilities. Thus, it is necessary to identify students less competent in English early during the undergraduate period and implement programmes to help them. Furthermore it might be necessary to conduct a formal English training programme for students for at least few months prior to entering medical school.

The Sedlacek’s Non-Cognitive questionnaire has demonstrated poor predictive value in first year medical students [11]. However we demonstrate that the ability to understand ones strengths and weakness (Self-appraisal), a positive self image (Self-concept), confidence, demonstration of leadership qualities and preference of long-term goals were all significant predictors of academic success in the final MBBS examination. The observed difference could be due to differences in; a) study settings, b) stage of assessment in the undergraduate period and c) method of assessment. Medical schools throughout the world use a variety of criteria to select applicants for admission. These criteria attempts to assess academic performance and personal characteristics suitable for a medical career. Although evaluating academic preparation is simple, assessing personal characteristics are difficult. Non-cognitive testing has been proposed as one such method to assess personal characteristics. However ‘non-cognitive’ tests at present are associated with numerous questions related to their validity, reliability, fairness, and cost. Therefore, before changing admission policies in ‘Traditional’ Sri Lankan medical schools by using non-cognitive tests, an open discussion among all stakeholders in the admissions process is critically important.

There are several limitations in the present study. Although we evaluated academic success, presently it is not known whether this academic success translates in to better clinical performance and improved patient outcomes. This could be a potential area of future research. Furthermore, we also did not evaluate the impact of non-cognitive characteristics on patient care. Our results show that students from Colombo have a higher academic success than students from other areas. However it would also be interesting to do a further evaluation of the few successful students who are not from Colombo to understand what factors influenced their success.

## Conclusion

Non-cognitive characteristics are important predictors of academic success among medical students. A combined system incorporating both past academic performance and non-cognitive characteristics might help improve the selection process and early recognition of Low achievers.

## Competing interests

The authors declare that they have no competing interests.

## Pre-publication history

The pre-publication history for this paper can be accessed here:

http://www.biomedcentral.com/1472-6920/12/66/prepub
